# Word Problem Solving in Contemporary Math Education: A Plea for Reading Comprehension Skills Training

**DOI:** 10.3389/fpsyg.2016.00191

**Published:** 2016-02-17

**Authors:** Anton J. H. Boonen, Björn B. de Koning, Jelle Jolles, Menno van der Schoot

**Affiliations:** Department of Pedagogical and Educational Sciences, Section of Educational Neuroscience, Faculty of Behavioral and Movement Sciences & LEARN! Research Institute for Learning and Education, Vrije Universiteit AmsterdamAmsterdam, Netherlands

**Keywords:** word problem solving, mental representation skills, reading comprehension skills, Realistic Math Education, consistency effect

## Abstract

Successfully solving mathematical word problems requires both mental representation skills and reading comprehension skills. In Realistic Math Education (RME), however, students primarily learn to apply the first of these skills (i.e., representational skills) in the context of word problem solving. Given this, it seems legitimate to assume that students from a RME curriculum experience difficulties when asked to solve semantically complex word problems. We investigated this assumption under 80 sixth grade students who were classified as successful and less successful word problem solvers based on a standardized mathematics test. To this end, students completed word problems that ask for both mental representation skills and reading comprehension skills. The results showed that even successful word problem solvers had a low performance on semantically complex word problems, despite adequate performance on semantically less complex word problems. Based on this study, we concluded that reading comprehension skills should be given a (more) prominent role during word problem solving instruction in RME.

## Introduction

In the last decades, mathematical word problem solving has gained much attention from both researchers and educational practitioners (Campbell, 1992; Hegarty et al., 1995; Hajer, 1996; Depaepe et al., 2010; Hickendorff, 2011, 2013; Moreno et al., 2011; Boonen et al., 2013; Swanson et al., 2013). Mathematical word problems refer to mathematical exercises that present relevant information on a problem as text, rather than in the form of mathematical notation (Rasmussen and King, 2000; Timmermans et al., 2007). Hence, effectively solving a mathematical word problem is assumed to depend not only on students’ ability to perform the required mathematical operations, but also on the extent to which they are able to accurately understand the text of the word problem (Lewis and Mayer, 1987; Hegarty et al., 1995; Van der Schoot et al., 2009; Jitendra and Star, 2012). Both of these aspects are related in such a way that developing a deeper understanding of the text of the word problem serves as a crucial step before the correct mathematical computations can be performed. Hence, a key challenge for word problem solvers is to get an adequate understanding of the problem statement (Lee et al., 2009; Thevenot, 2010; Boonen et al., 2013).

Two individual skills are relevant in this regard. First, an important factor contributing to a deeper understanding of the text of the word problem is the ability to construct a rich and coherent mental representation containing all (the relations between the) solution-relevant elements that are derived from the text base of the word problem (De Corte et al., 1985; Hegarty et al., 1995; Pape, 2003). That is, word problem solvers have to use a problem-model strategy in which they translate the problem statement into a qualitative mental representation of the problem situation hidden in the text (Pape, 2003; Van der Schoot et al., 2009). This mental representation subsequently allows them to make a solution plan and execute the required mathematical operations. Although successful word problem solvers appear to employ such a problem-model strategy by drawing on their mental representation skills, less successful problem solvers often adopt an impulsive, superficial direct translation strategy, in which they only focus on selecting the presented numbers that, in turn, form the basis for their mathematical calculations (Verschaffel et al., 1992; Hegarty et al., 1995).

The second important individual skill in word problem solving success substantiated by research evidence is the influence of a student’s reading comprehension abilities (Pape, 2003; Van der Schoot et al., 2009; Boonen et al., 2013). It has been suggested that reading comprehension abilities are especially helpful in dealing with semantic-linguistic word problem characteristics such as the sequence of the known elements in the text of the word problem, the degree to which the semantic relations between the given and unknown quantities of the problem are made explicit, and the relevance of the information in the text of the word problem (De Corte et al., 1985, 1990; Verschaffel et al., 1992; Marzocchi et al., 2002).

Moreover, reading comprehension skills appear to be more important in overcoming such textual complexities than being able to use one’s mental representation skills (De Corte et al., 1985, 1990). This might explain why the use of a problem-model strategy is not sufficient in all circumstances. That is, word problems containing semantically complex features require both accurate mental representation skills and reading comprehension skills, whereas for word problems with a lower semantic-linguistic complexity, well-developed mental representational skills might be sufficient.

These findings suggest that, to teach students how to effectively solve mathematical word problems, mental representation skills and reading comprehension skills should both be part of the mathematics education program. Particularly, paying attention to semantic-linguistic features of word problems is relevant to help students improve their word problem solving success, as word problems become semantically more complex as students progress in their educational career, for example, when they make the transition to secondary education. Word problems offered in secondary school subjects like geometry, physics and biology, include more verbal information and generally contain more complex semantic-linguistic text features (Silver and Cai, 1996; Helwig et al., 1999).

The Netherlands, like many other countries, currently places great emphasis on the teaching of word problem solving in contemporary mathematics education (Ruijssenaars et al., 2004; Elia et al., 2009). The teaching of mathematics in the Netherlands takes place within the context of a domain-specific instructional approach, called Realistic Mathematics Education (RME, Van den Heuvel-Panhuizen, 2003), where the process of mathematical word problem solving plays an important role (Van den Boer, 2003; Barnes, 2005; Prenger, 2005; Van den Heuvel-Panhuizen, 2005; Hickendorff, 2011). Studies investigating the educational practice of RME show that the teaching of mental representation skills receives much attention in word problem solving instruction (Van den Heuvel-Panhuizen, 2003; Van Dijk et al., 2003; Elia et al., 2009). However, reading comprehension skills enabling students to become sensitive to semantic-linguistic complexities in a word problem appear to be trained fewer and less explicitly in the instructional practice of RME, in spite of its proven importance in previous studies (e.g., De Corte et al., 1985, 1990; Hegarty et al., 1992). This is presumably because teachers may underestimate or are not aware of the importance of reading comprehension skills for solving word problems (Hajer, 1996; Van Eerde, 2009). Thus, the current approach to teaching word problem solving appears to emphasize the development of mental representation skills, but seems to pay less attention to the role of reading comprehension skills. In this respect, the way in which word problem solving is taught in the RME curriculum does not seem to be aligned with what is currently known from research about the factors involved in effective word problem solving.

Based on the above analysis of the RME curriculum it seems legitimate to assume that students attending such a curriculum may be at a disadvantage when semantic-linguistic characteristics of a word problem have to be taken into account. That is, students from an RME curriculum are likely to experience difficulties when ask to solve mathematical word problems with a high semantic-linguistic complexity. To test this assumption, we compared students’ performance on word problems obtained while following the RME curriculum to their performances on an independent word problem solving task. First, we classified students as successful or less successful word problem solvers with the help of a mathematics test that is part of the RME curriculum, viz., the CITO Mathematics test. This test can be considered a method-specific (i.e., RME-specific) mathematics test of students’ word problem solving performance, as it builds upon the currently used instructional method for word problem solving. Hence, this test reflects the skills that students learn in the RME classroom, in order to solve word problems (Doorman et al., 2007; Hickendorff, 2011). Second, we examined students’ performance on an independent word problem solving test, which contained either word problems that they could solve by only using their mental representation skills, or word problems that required them to also rely on their reading comprehension skills for handling semantic-linguistic complexities in the word problems. This procedure provides an advantage over prior studies of, among others, Hegarty et al. (1995), Pape (2003), and Van der Schoot et al. (2009), which typically used the main dependent variable of the study (i.e., problem solving success) as an outcome measure as well as a means to classify students into successful and less successful word problem solvers. The classification used in the present study, on the other hand, is based on an external, well-established measure of mathematical word problem solving, which is independent of the main dependent variable of the study (i.e., word problem solving success). This allowed us to make more meaningful group comparisons.

As previously mentioned, a key aspect that differentiates successful from less successful word problem solvers concerns their ability to construct an accurate mental representation of the problem text. Previous studies have shown that asking students to solve compare problems, especially inconsistent compare problems (see Example 1), is a suitable method for investigating whether or not they have effectively constructed an accurate mental representation of the problem statement (e.g., Pape, 2003; Van der Schoot et al., 2009).

[Example 1 – inconsistent word problem]

At the grocery store, a bottle of olive oil costs 7 euro.

That is 2 euro *more than* at the supermarket.

If you need to buy seven bottles of olive oil, how much will it cost at the supermarket?

[Example 2 – consistent word problem]

At the grocery store, a bottle of olive oil costs 7 euro.

At the supermarket, a bottle of olive oil costs 2 euro *more than* at the grocery store.

If you need to buy 7 bottles of olive oil, how much will you pay at the supermarket?

In inconsistent word problems like the one presented in Example 1, the translation process requires the identification of the pronominal reference ‘that is’ as the indicator of the relation between the value of the first variable (‘the price of a bottle of olive oil at the grocery store’) to the second (‘the price of a bottle of olive oil at the supermarket’). This identification is necessary to become cognizant of the fact that, in an inconsistent compare problem, the relational term ‘more than’ refers to a subtraction operation rather than to an addition operation. So, inconsistent word problems create greater cognitive complexity than consistent word problems (see Example 2), requiring students to ignore the well-established association between *more* with increases and addition, and *less* with decreases and subtraction (Schumacher and Fuchs, 2012). Empirical evidence corroborates this interpretation by showing that word problem solvers make more (reversal) errors on inconsistent than on consistent word problems (i.e., consistency effect, Lewis and Mayer, 1987; Pape, 2003; Van der Schoot et al., 2009). Especially students who fail to build an accurate mental representation of the problem statement, and thus immediately start calculating with the given numbers and relational term, seem to be less successful on inconsistent word problems (Hegarty et al., 1995).

In the present study, we expected neither successful nor less successful problem solvers to experience difficulties with solving consistent compare word problems. However, we did assume that successful word problem solvers in the RME curriculum would experience less difficulties with correctly solving inconsistent compare problems as a result of their reliance on mental representation skills (acquired during word problem solving instruction in RME), than less successful problem solvers who employ a more superficial problem solving approach (Verschaffel et al., 1992; Van der Schoot et al., 2009).

It is important to keep in mind that this only holds for consistent and inconsistent compare problems with a low semantic complexity; that is, problems that only tap into students’ ability to construct an accurate mental representation. If the semantic complexity of compare problems increases, we expected that even students classified as successful word problem solvers (according to our classification based on the RME instruction) may come to experience difficulties with correctly solving inconsistent compare problems. In this case, correctly solving a word problem requires students to use both mental representational skills and reading comprehension skills, while word problem solving instruction in RME (presumably) has provided students only with considerable training in the first of these two skills.

A relatively well-studied and accepted way to increase the semantic complexity of (inconsistent) compare problems is to manipulate the relational term (Lewis and Mayer, 1987; Van der Schoot et al., 2009). According to the lexical marking principle (Clark, 1969), it is more difficult to process marked relations terms (such as ‘less’ in the antonym pair ‘more-less,’ ‘narrow’ in ‘wide-narrow’ or ‘short’ in ‘tall-short’) than unmarked relational terms (e.g., more, wide, tall). Consistent with this, research has shown that students find it easier to convert the unmarked relational term ‘more than’ into a subtraction operation than the marked relational term ‘less than’ into an addition operation (Clark, 1969; Lewis and Mayer, 1987; Kintsch, 1998; Pape, 2003; Van der Schoot et al., 2009). In the present study, we therefore refer to word problems containing a marked relational term (‘more than’) as semantically more complex word problems, whereas word problems with an unmarked relational term (‘less than’) are referred to as semantically less complex word problems (see Examples 3 and 4 for examples of marked and unmarked word problems respectively). Importantly, the difficulties experienced with solving marked inconsistent word problems lie in the fact that these problems draw on students’ use of their mental representation skills as well as on their reading comprehension skills. Accordingly, the influence of reading comprehension skills on word problem solving can only be studied for students who mentally represent the problem statement accurately, that is, the group of successful problem solvers in our study. So, although our group of successful word problem solvers may draw upon their mental representation skills, the insufficient attention to reading comprehension skills in the educational practice of RME is likely to cause them to experience difficulties with correctly solving (semantically complex) marked inconsistent word problems.

[Example 3 – marked word problem]

At the grocery store, a bottle of olive oil costs 7 euro.

At the supermarket, a bottle of olive oil costs 2 euro *less than* at the grocery store.

If you need to buy seven bottles of olive oil, how much will you pay at the supermarket?

[Example 4 – unmarked word problem]

At the grocery store, a bottle of olive oil costs 7 euro.

That is 2 euro *less than* at the supermarket.

If you need to buy seven bottles of olive oil, how much will it cost at the supermarket?

According to several researchers, the extent to which successful word problem solvers might be able to overcome difficulties with correctly solving marked inconsistent word problems is related to their reading comprehension skills (e.g., Lee et al., 2004; Van der Schoot et al., 2009). Translating a marked relational term like ‘less than’ into an addition operation is found to be closely associated with general measures of reading comprehension (Lee et al., 2004; Van der Schoot et al., 2009). This suggests that reading comprehension skills, together with mental representation skills, might be necessary to deal with semantically complex word problems. The present study therefore also takes into account students’ general reading comprehension ability.

In sum, the present study aimed to test the following hypotheses:

1. We hypothesized that, as a result of difficulties with constructing a coherent mental representation of word problems, less successful word problem solvers in the RME curriculum would make more errors on both unmarked and marked inconsistent word problems than on unmarked and marked consistent word problems.
2. We hypothesized that, as a result of paying insufficient attention to reading comprehension skills in the teaching of word problem solving, successful word problem solvers in the RME curriculum would experience difficulties with solving semantically complex, marked inconsistent word problems, but not with solving semantically less complex, unmarked, inconsistent word problems.
3. We hypothesized that, as a result of the alleged relation between reading comprehension ability and the ability to overcome the semantic-linguistic complexities of a word problem, a positive relation for successful problem solvers exists between reading comprehension ability and the number of correctly solved marked inconsistent word problems.

## Materials and Methods

### Selection of Participants

Data from 80 Dutch sixth-grade students (42 boys, 38 girls) from eight elementary schools in the Netherlands were collected. These students had a mean age of 11.72 years (*SD* = 0.40). They were almost equally divided in two groups (by means of the median split method) on the basis of their score on the CITO (Institute for Educational Measurement) Mathematics test (2008). This selection procedure resulted in a group of less successful word problem solvers (*N* = 41) and a group of successful word problems solvers (*N* = 39). The CITO Mathematics test is a nationwide standardized test that reflects the way in which word problem solving is instructed in Realistic Mathematics Education. The test contains elements like *mental arithmetic* (addition, subtraction, multiplication, and division), *complex applications* (problems involving multiple operations) and *measurement and geometry* (knowledge of measurement situations), all of which are offered as mathematical word problems. The internal consistency of this test was high (Cronbach’s α = 0.95, Janssen et al., 2010).

Parents provided written informed consent based on printed information about the purpose of the study. This study was carried out in accordance with the ethical procedures of the Vrije Universiteit Amsterdam.

### Instruments and Procedure

The two measurement instruments that were used in this study were administrated to the students by three trained independent research assistants in a session of approximately 45 min.

#### Inconsistency Task

The inconsistency task contained eight two-step compare problems (see Appendix in Supplementary Material) that were selected from the study of Hegarty et al. (1992) and were translated into Dutch. All of the word problems consisted of three sentences. The first sentence of each compare problem was an assignment statement expressing the value of the first variable, namely the price of a product at a well-known Dutch store or supermarket (e.g., At Aldi a bottle of wine costs 4 euro). The second sentence contained a relational statement, expressing the value of the second variable (i.e., the price of this product at another store or supermarket) in relation to the first (e.g., At Boni, a bottle of wine costs 3 euro more than at Aldi). In the third sentence, the problem solver was asked to find a multiple of the value of the second variable (e.g., If you need to buy three bottles of wine, how much will you pay at Boni?). The answer to these compare problems always involved first computing the value of the second variable (e.g., 4 + 3 = 7), and then multiplying this solution by the quantity given in the third sentence (e.g., 7 times 3 = 21).

The eight compare problems were separated in four different word problem types (see Appendix in Supplementary Material) by crossing the following two within-subject factors: *Consistency* (consistent vs. inconsistent) and *Markedness* (unmarked vs. marked). Consistency referred to whether the relational term in the second sentence was consistent or inconsistent with the required arithmetic operation. A consistent sentence explicitly expressed the value of the second variable (e.g., At Boni a bottle of wine costs 3 euro [more/less] than at Aldi) introduced in the prior sentence (e.g., At Aldi a bottle of wine costs 4 euro). An inconsistent sentence related the value of the second variable to the first by using a pronominal reference (e.g., That is 3 euro [more/less] than at Aldi). Consequently, the relational term in a consistent compare problem primed the appropriate arithmetic operation (‘more than’ when the required operation is addition, and ‘less than’ when the required operation is subtraction). The relational term in an inconsistent compare problem primed the inappropriate arithmetic operation (‘more than’ when the required operation is subtraction, and ‘less than’ when the required operation is addition). Markedness referred to whether the relational term was a marked (i.e., less than) or an unmarked (i.e., more than) member of the antonym pair ‘more-less.’ As mentioned earlier, markedness was used to manipulate the semantic complexity of the relational term. A marked relational term (i.e., less than) is semantically more complex than an unmarked relational term (i.e., more than). Hence, marked and unmarked word problems were considered as semantically more complex and semantically less complex word problems respectively.

The stimuli were arranged in four material sets. Each participant was presented with eight word problems, two from each word problem type. The order in which the word problems were presented in each set was pseudorandomized. Each set was presented to 20 participants. Across sets and across participants, each word problem occurred equally often in the unmarked/consistent, marked/consistent, unmarked/inconsistent and marked/ inconsistent version to ensure full combination of conditions and materials. Across word problems, we controlled for the difficulty of the required calculations, and for the number of letters in the names of the variables (i.e., stores) and products. To ensure that the execution of the required arithmetic operations would not be a determining factor in students’ word problem solving performance, the operations were selected on the basis of the following rules: (1) the answers to the first step of the operation were below 10; (2) the final answers were between 14 and 40; (3) none of the first steps or final answers contained a fraction of a number or negative number; (4) no numerical value occurred twice in the same problem; and (5) none of the (possible) answers were 1. The numerical values used in consistent and inconsistent problems of each word problem type were matched for magnitude (see Van der Schoot et al., 2009).

For the analyses, we looked at students’ accuracy (i.e., the amount of correct answers) on each of the four word problem types: (1) unmarked/consistent; (2) marked/consistent; (3) unmarked/inconsistent; and (4) marked/inconsistent. The internal consistency of this measure in the present study was high (Cronbach’s α = 0.90).

#### Reading Comprehension

The (Grade 6 version of the) normed standardized CITO (Institute for Educational Measurement) Test for Reading Comprehension (2010) of the Dutch National Institute for Educational Measurement was used to assess children’s reading comprehension level. This test is part of the standard Dutch CITO pupil monitoring system and is designed to determine general reading comprehension level in elementary school children. This test consists of two modules, each involving a text and 25 multiple choice questions. The questions pertained to the word, sentence or text level, and tapped both the text base and situational representation that the reader constructed from the text (Kintsch, 1998). On this test, children’s reading comprehension level is expressed by a reading proficiency score, which, in this study, ranged from 15 to 95 (*M* = 40.51, *SD* = 13.94). The internal consistency of this test was high with a Cronbach’s alpha of 0.89 (Weekers et al., 2011).

### Data Analysis

A 2 × 2 × 2 analysis of variance (ANOVA) was conducted with Consistency (consistent vs. inconsistent) and Markedness (unmarked vs. marked) as within-subject factors and Group (less successful vs. successful word problem solvers) as the between-subject factor. Follow-up tests were performed using paired sample *t*-tests. The partial eta-squared ($\eta_{p}^{2}$) was calculated as a measure of effect size (Pierce et al., 2004). According to Pierce et al. (2004), values of 0.02, 0.13, and 0.26 represent small, medium, and large effect sizes respectively.

In the present study, the role of reading comprehension in the four word problem types was examined by calculating the product-moment correlations (Pearson’s *r*) between reading comprehension and the difference score between the unmarked inconsistent and consistent word problem types, and the correlation between reading comprehension and the difference score between the marked inconsistent and consistent word problem types. These difference scores reflect the differences in performance between the consistent and inconsistent word problem types, and can be taken as a measure of the extent to which students are able to construct a mental representation of the described problem situation. The lower the difference score, the less word problem solvers suffer from the inconsistency. The correlations were first calculated for the less successful and successful word problem solvers together, and then, to test the third hypothesis, for each of these groups separately.

Our approach deviates from, but provides an important advantage over, the study by Van der Schoot et al. (2009), who added reading comprehension as a covariate in the repeated measures ANOVA. That is, the results obtained by Van der Schoot et al. (2009) could provide only limited insight into the exact locus of the covariate’s effect, as it was not known which group (less successful or successful word problem solvers) or in which word problem type (consistent unmarked/marked or inconsistent unmarked/marked) reading comprehension played a role. Moreover, it turns out that the repeated measures ANCOVA does change the main effects of the repeated measures compared to assessing the main effects via a simple repeated measures ANOVA (see Thomas et al., 2009). So, the approach used in the present study enabled us to obtain more specific insight into the precise role of reading comprehension in word problem solving. In all analyses an alpha of 0.05 was used to test the significance of the results.

## Results

The overall means (*M*) and standard deviations (*SD*) for the main factors in this study, as well as their intercorrelations, are displayed in **Table 1**. As can be seen, there was a significant main effect of Consistency [*F*(1,78) = 23.84, *p* = 0.00, $\eta_{p}^{2}$ = 0.23], indicating that consistent word problems were completed more accurately than inconsistent word problems (i.e., consistency effect). There was no significant main effect of Markedness [*F*(1,78) = 2.64, *p* = 0.11], suggesting that overall not more errors were made on marked than on unmarked word problems. The main effect of Group was also not significant [(1,78) = 1.15, *p* = 0.29)], indicating that overall successful problem solvers did not show a higher problem solving performance than less successful problem solvers.

**Table 1:** Overall means, standard deviations, and correlations of the main variables.

	*M*	*SD*	1	2	3	4	5	6
(1) Reading comprehension	40.51	13.94	–					
(2) Mathematics (RME) test	89.25	21.94	0.59^∗∗^	–				
(3) Consistent	3.60	0.72	0.21	0.33^∗∗^	–			
(4) Inconsistent	2.96	1.24	0.34^∗∗^	0.27^∗^	0.39^∗∗^	–		
(5) Unmarked	3.36	0.85	0.24^∗^	0.24^∗^	0.61^∗∗^	0.79^∗∗^	–	
(6) Marked	3.20	1.04	0.35^∗∗^	0.35^∗∗^	0.67^∗∗^	0.83^∗∗^	0.55^∗∗^	–
(7) Successful problem solvers	6.76	1.46	0.38^∗^	0.54^∗∗^	0.57^∗∗^	0.96^∗∗^	0.85^∗∗^	0.85^∗∗^
(8) Less successful problem solvers	6.36	1.84	0.31	0.38^∗^	0.81^∗∗^	0.90^∗∗^	0.87^∗∗^	0.94^∗∗^

*^∗^p < 0.05, ^∗∗^p < 0.001.*

**FIGURE 1:**
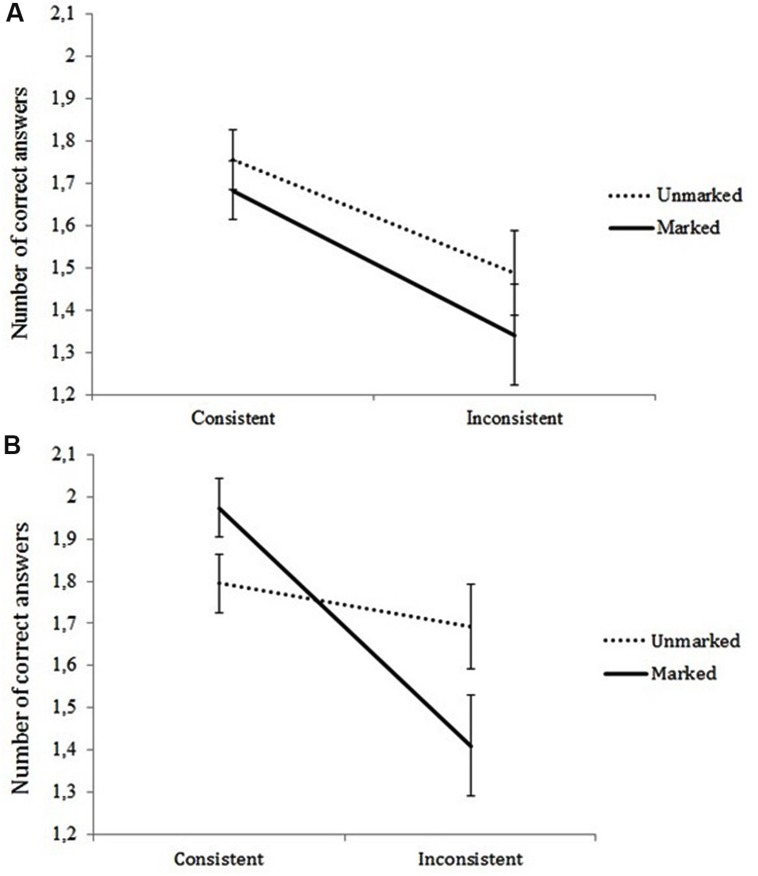
**Performance on the four types of word problems for the less successful (A) and successful problem solvers (B)**.

Regarding the interacting effects between Consistency and Markedness, the analysis revealed a significant interaction [*F*(1,78) = 7.64, *p* = 0.01, $\eta_{p}^{2}$ = 0.09] showing that overall the consistency effect was present for marked word problems but absent for unmarked word problems. Of more interest, in light of our hypotheses, is that, as expected, the Consistency × Markedness interaction differed for less successful and successful word problem solvers. This was evidenced by a significant three-way interaction between Consistency, Markedness, and Group [*F*(1,78) = 4.32, *p* = 0.03, $\eta_{p}^{2}$ = 0.05]. In **Figure 1**, word problem solving performance is presented as a function of consistency (consistent vs. inconsistent) and markedness (marked vs. unmarked) for less successful problem solvers (**Figure 1A**), and for successful problem solvers (**Figure 1B**), respectively.

As shown in **Figure 1A**, the main effect of Consistency [*F*(1,38) = 8.16, *p* = 0.01, $\eta_{p}^{2}$ = 0.18] indicates that less successful word problem solvers showed the consistency effect. Given the non-significant Consistency × Markedness interaction [*F*(1,38) = 0.25, *p* = 0.62], the consistency effect was present for both marked and unmarked word problems. No significant main effect of Markedness was found [*F*(1,38) = 0.12, *p* = 0.74]. So, less successful word problem solvers performed significantly lower on both the unmarked and marked inconsistent word problem types, compared to the consistent unmarked and marked word problem types [*t*(38) = 1.86, *p* = 0.04; *t*(38) = 2.57, *p* = 0.01 respectively].

As can be seen in **Figure 1B**, the group of successful problem solvers resembled the less successful problem solvers in that there was a main effect of Consistency [*F*(1,40) = 16.29, *p* = 0.00, $\eta_{p}^{2}$ = 0.29], but no significant main effect of Markedness [*F*(1,40) = 0.27, *p* = 0.61]. However, in contrast to the group of less successful problem solvers, the consistency effect in the group of successful problem solvers was present for marked but absent for unmarked word problems [Consistency × Markedness interaction: *F*(1,40) = 17.44, *p* = 0.00, $\eta_{p}^{2}$ = 0.30]. This indicates that successful word problem solvers performed significantly lower on marked inconsistent compared to marked consistent word problems [*t*(40) = 5.07, *p* = 0.00], whereas performance on unmarked consistent and unmarked inconsistent word problem types did not differ significantly [*t*(40) = 1.52, *p* = 0.13].

In sum, these findings show that less successful word problem solvers demonstrated the consistency effect on both semantic-linguistically simple (i.e., unmarked) and complex (i.e., marked) word problems, whereas successful word problem solvers only demonstrated the consistency effect when the word problem text contained complex semantic-linguistic features (i.e., marked).

Regarding the role of reading comprehension skills in word problem solving the following findings were obtained. Overall, there was a significant correlation between reading comprehension and mathematics scores obtained from the curriculum-specific RME test (*r* = 0.59, *p* = 0.00). This suggests that students with higher reading comprehension scores also showed higher scores on the RME mathematics test. To obtain more detailed insight into the role of reading comprehension skills in solving marked and unmarked word problems, reading comprehension scores were correlated with the difference scores (inconsistent – consistent) computed for the marked and unmarked word problem types. Results showed that reading comprehension was significantly correlated with the difference score for unmarked word problems (*r* = 0.19, *p* = 0.04) and had a marginally significant correlation with the difference score for marked word problems (*r* = 0.17, *p* = 0.06). This suggests that overall reading comprehension abilities are relevant to solving both marked and unmarked word problems.

When looking at the successful and less successful problem solvers separately, the results showed, similar to the overall findings, that reading comprehension was significantly correlated with the scores on the RME-specific mathematics test for both successful (*r* = 0.48, *p* = 0.00) and less successful problem solvers (*r* = 0.64, *p* = 0.00). So, for successful and less successful problem solvers higher reading comprehension abilities were associated with higher RME mathematics scores. Furthermore, successful word problem solvers (*M* = 46.42, *SD* = 2.66) scored significantly higher on the standardized reading comprehension test than less successful word problem solvers (*M* = 35.02, *SD* = 1.27) [*t*(53.32) = 3.87, *p* = 0.00].

More specific analyses focusing on the hypothesized relation between reading comprehension skills and solving marked inconsistent word problems revealed the following pattern of findings. In line with our expectations, the results of the correlational analyses between reading comprehension and the difference scores for marked and unmarked word problems showed that only in the group of successful word problem solvers the difference score for the marked word problem type was significantly related to reading comprehension (*r* = -0.40, *p* = 0.01). Importantly, reading comprehension was not correlated with the successful word problem solvers’ difference scores for unmarked word problems (*r* = -0.27, *p* = 0.10). Furthermore, in the group of less successful word problem solvers, reading comprehension was also not correlated with the difference scores computed for either unmarked (*r* = -0.04, *p* = 0.76) or marked word problems (*r* = -0.04, *p* = 0.83).

So, only in the group of successful word problem solvers, a higher reading comprehension score was associated with a smaller difference score. That is, the vulnerability for the consistency effect on marked word problems was lower for students who have higher reading comprehension abilities. This suggests that students with higher reading comprehension abilities appear to suffer less from being primed to an inconsistent arithmetic operation (i.e., being directed toward a subtraction operation by ‘less than’ while addition is required) in solving marked inconsistent word problems.

## Discussion

This study was motivated by the observation that contemporary RME primarily teaches students to use their mental representation skills, and focuses much less on using reading comprehension skills, to solve mathematical word problems. Against this background, we set out to investigate the assumption that students from an RME curriculum experience difficulties when having to solve mathematical word problems that are semantic-linguistically complex. We therefore designed a study in which we not only manipulated the extent to which mental representation skills were required, but also varied the semantic complexity of the word problems by using a marked (i.e., high semantic complexity) or unmarked (i.e., low semantic complexity) relational term in the word problem text. Moreover, we classified students as successful and less successful word problem solvers on the basis of their performance on an independent and well-established RME-specific mathematics test.

Using this classification procedure, it was hypothesized that less successful word problem solvers would experience difficulties with correctly solving inconsistent word problems irrespective of their semantic complexity (Hypothesis 1). This hypothesis was confirmed by our analyses, which showed that less successful word problem solvers performed poorly on both marked and unmarked inconsistent word problems. Successful word problem solvers, on the other hand, were able to effectively solve inconsistent word problems that had a low semantic complexity. So, these findings show that the RME-based classification in successful and less successful problem solvers was also reflected in our experimental word problem solving task.

However, on semantically complex word problems even the successful problem solvers experienced difficulties, as indicated by the large number of errors they made on marked inconsistent word problems (Hypothesis 2). More concretely, successful word problem solvers found it more difficult to translate a marked relational term (‘less than’) into an addition operation, than to translate an unmarked relational term (‘more than’) into a subtraction operation.

These findings once again support prior observations that (subtle) semantic-linguistic elements of a word problem, more specifically the marked relational term, influence word problem solving success (Clark, 1969; Lewis and Mayer, 1987; Kintsch, 1998; Pape, 2003; Van der Schoot et al., 2009). Moreover, they are in line with empirical work consistently reporting processing problems with marked terms, which are suggested to be caused by the semantic representation of negative poles of antonym pairs (e.g., more than vs. less than) like ‘less than’ being more fixed and complex, and therefore less likely to be reversed, than that of the positive poles like ‘more than’ (e.g., Lewis and Mayer, 1987; for a detailed explanation of the underlying mechanism, see, e.g., Clark, 1969). For example, earlier studies have shown that students are less able to recall marked terms accurately in memory tasks (Clark and Card, 1969), have slower naming responses for marked terms in naming tasks (Schriefers, 1990), have slower solution times for problems with marked adjectives in reasoning problems (French, 1979), and, the finding replicated in this study, experience problems with reversing a marked inconsistent word problem (e.g., Pape, 2003; Van der Schoot et al., 2009).

Importantly, our results reveal the interesting situation that students classified as successful word problem solvers in an RME curriculum are unsuccessful in solving semantically complex (inconsistent) word problems. The fact that successful problem solvers were able to solve inconsistent word problems with a lower semantic complexity suggests that this poor performance on semantically complex word problems is not due to shortcomings in their mental representation skills. Rather, it seems that successful problem solvers particularly have difficulties to effectively handle semantic-linguistic complexities in word problems. This suggests that students lack the reading comprehension skills required for identifying and translating a primed mathematical operation to the ‘word problem appropriate’ mathematical operation. In the case of marked inconsistent word problems, this means that even successful students find it difficult to convert ‘less than’ into an addition operation. Although it could be argued that this is likely the result of the relatively little attention to the development of reading comprehension skills in the context of mathematical word problem solving in RME (e.g., Elia et al., 2009), this speculative interpretation needs to be further substantiated in future research.

Building upon prior studies (e.g., Lee et al., 2004; Van der Schoot et al., 2009), another aim of this study was to investigate whether reading comprehension skills could help (successful) word problem solvers to overcome the semantically complex marked relational term in an inconsistent word problem. In line with our expectations, reading comprehension was positively related to the performance on marked (but not unmarked) inconsistent word problems for the group of successful word problem solvers, whereas for the less successful group no significant relations were found between reading comprehension and word problem solving (Hypothesis 3).

These results provide corroborating evidence that general reading comprehension skills play an important role in students’ ability to correctly solve semantically complex word problems. Moreover, our findings represent an advance over prior work by more specifically delineating which types of word problems and for which students reading comprehension ability might have an effect. This study shows that reading comprehension skills are especially helpful when it comes to improving the performance on semantically complex word problems by successful word problem solvers (as classified by the RME mathematics test). More specifically, reading comprehension skills are relevant for word problem solving primarily in helping students to effectively translate complex (i.e., marked) relations terms encountered in inconsistent word problems to the correct mathematical operation (i.e., addition). From this, it is evident that reading comprehension skills provide a valuable addition to mental representation skills for word problem solving, and that simply relying on mental representation skills is not sufficient to correctly solve semantically complex word problems. This suggests that in addition to teaching students to use their mental representation skills to solve word problems, word problem solving instruction should have sufficient attention for developing and using reading comprehension skills related to identifying and dealing with semantic-linguistic features in the word problem statement.

It is important to start developing such skills early in elementary school, as word problems get semantically more complex when students progress in their educational career, for example when making the transition from elementary to secondary education (Silver and Cai, 1996; Helwig et al., 1999). Particularly in instructional approaches focused on word problem solving that show an imbalance between the amount of instruction time being devoted to the teaching of mental representation skills and reading comprehension skills, such as in RME, it is important to make teachers aware of this unequal distribution. Encouraging them to pay more attention to reading comprehension skills and teaching students how to deal with semantic-linguistic characteristics in word problems would then provide a good starting point to work toward more equally balanced word problem solving instructions. Moreover, it is useful to make a distinction between learning to process more subtle semantic-linguistic text features (like a marked relation term) and dealing with more general semantic text complexities (like the relevance of the information in the word problem text, the explicitness of the described relations, and the sequence of the known elements in the word problem text).

These and other practical aspects of the results, such as finding the optimal balance between the amount of instruction in strategic mental representation skills and reading comprehension skills, remain to be addressed in future research. Presumably, currently effective intervention programs that focus on both strategic mental representation skills and reading comprehension skills, such as schema-based instruction (e.g., Jitendra et al., 2002, 2011), and the *Solve It!* instruction method (Montague et al., 2000; Krawec et al., 2013), could provide a fruitful starting point in pursuing this challenge.

## Author Contributions

All authors listed, have made substantial, direct and intellectual contribution to the work, and approved it for publication.

## Conflict of Interest Statement

The authors declare that the research was conducted in the absence of any commercial or financial relationships that could be construed as a potential conflict of interest.
