# Supplementary material for: Word Problem Solving in Contemporary Math Education: A Plea for Reading Comprehension Skills Training
Source: Front Psychol. 2016 Feb 17;7:191. doi: 10.3389/fpsyg.2016.00191 (PMC4756284; doi:10.3389/fpsyg.2016.00191)
Supplement: Supplementary file 1 [file Appendix.DOCX]

**Appendix**

The following eight word problems were used in this study. The four word problem types resulted from crossing the factors consistency (consistent vs. inconsistent) and markedness (unmarked vs. marked).

| **Word problem type** |  |  |
| --- | --- | --- |
| Consistent Unmarked | At Albert Heijn, a piece of cheese costs 4 euro.  At the cheese shop, a piece of cheese costs 3 euro more than at Albert Heijn.  If you need to buy 3 pieces of cheese, how much will you pay at the cheese shop?  Answer: 4 + 3 = 7, 7 x 3 = 21 | At Jumbo, a pack of washing powder costs 5 euro.  At Spar a pack of washing powder costs 3 euro more than at Jumbo.  If you need to buy 4 packs of washing powder, how much will you pay at Spar?  Answer: 5 + 3 = 8, 8 x 4 = 32 |
| Consistent Marked | At the grocery store, a bottle of olive oil costs 9 euro.  At the supermarket, a bottle of olive oil costs 4 euro less than at the grocery store.  If you need to buy 7 bottles of olive oil, how much will you pay at the supermarket?  Answer: 9 – 4 = 5, 5 x 7 = 35 | At the greengrocer, a bag of peanuts costs 9 euro.  At the convenience store, a bag of peanuts costs 4 euro less than at the greengrocer.  If you need to buy 5 bags of peanuts, how much will you pay at the convenience store?  Answer: 9 – 4 = 5, 5 x 5 = 25 |
| Inconsistent Unmarked | At Edah, a pack of candles costs 6 euro.  That is 2 euro more than at Super de Boer.  If you need to buy 6 pack of candles, how much will you pay at Super de Boer?  Answer: 6 – 2 = 4, 4 x 6 = 24 | At the bakery, a box of donuts costs 6 euro.  That is 3 euro more than at Subway.  If you need to buy 6 boxes of donuts, how much will you pay at Subway?  Answer: 6 – 3 = 3, 3 x 6 = 18 |
| Inconsistent Marked | At Etos, a bag of candy costs 6 euro.  That is 2 euro less than at Hema.  If you need to buy 3 bags of candy, how much will you pay at Hema?  Answer: 6 + 2 = 8, 8 x 3 = 24 | At Lidl, a bottle of syrup costs 5 euro.  That is 3 euro less than at Konmar.  If you need to buy 4 bottles of syrup, how much will you pay at Konmar?  Answer: 5 + 3 = 8, 8 x 4 = 32 |
